# Genome-wide organization and expression profiling of the R2R3-MYB transcription factor family in pineapple (*Ananas comosus*)

**DOI:** 10.1186/s12864-017-3896-y

**Published:** 2017-07-01

**Authors:** Chaoyang Liu, Tao Xie, Chenjie Chen, Aiping Luan, Jianmei Long, Chuhao Li, Yaqi Ding, Yehua He

**Affiliations:** 1 College of Horticulture, South China Agricultural University, Guangzhou, 510642 Guangdong China; 2 Key Laboratory of Horticultural Plant Biology of MOE (Ministry of Education), Huazhong Agricultural University, Wuhan, 430070 Hubei China

**Keywords:** Pineapple, Genome-wide, MYB transcription factor, Collinearity, Abiotic stress

## Abstract

**Background:**

The MYB proteins comprise one of the largest families of plant transcription factors, which are involved in various plant physiological and biochemical processes. Pineapple (*Ananas comosus*) is one of three most important tropical fruits worldwide. The completion of pineapple genome sequencing provides a great opportunity to investigate the organization and evolutionary traits of pineapple MYB genes at the genome-wide level.

**Results:**

In the present study, a total of 94 pineapple R2R3-MYB genes were identified and further phylogenetically classified into 26 subfamilies, as supported by the conserved gene structures and motif composition. Collinearity analysis indicated that the segmental duplication events played a crucial role in the expansion of pineapple MYB gene family. Further comparative phylogenetic analysis suggested that there have been functional divergences of MYB gene family during plant evolution. RNA-seq data from different tissues and developmental stages revealed distinct temporal and spatial expression profiles of the *AcMYB* genes. Further quantitative expression analysis showed the specific expression patterns of the selected putative stress-related *AcMYB* genes in response to distinct abiotic stress and hormonal treatments. The comprehensive expression analysis of the pineapple MYB genes, especially the tissue-preferential and stress-responsive genes, could provide valuable clues for further function characterization.

**Conclusions:**

In this work, we systematically identified *AcMYB* genes by analyzing the pineapple genome sequence using a set of bioinformatics approaches. Our findings provide a global insight into the organization, phylogeny and expression patterns of the pineapple R2R3-MYB genes, and hence contribute to the greater understanding of their biological roles in pineapple.

**Electronic supplementary material:**

The online version of this article (doi:10.1186/s12864-017-3896-y) contains supplementary material, which is available to authorized users.

## Background

Transcription factors play an important role in the regulation of gene transcription, and control many aspects of plant growth and development through activating or suppressing their target genes [1]. Regularly, transcription factors are encoded by multigene families. The MYB transcription factors are broadly dispersed in higher plants genomes, and comprise one of the largest transcription factor families in plants [2]. The MYB gene family members bear a characteristic N-terminal MYB DNA-binding domain, which is highly conserved among plants. The MYB domain usually contains 1–4 imperfect repeats (named R1, R2, R3 and R4), each with about 52 amino acid residues and forming three α-helices [3]. The second and third helices form a helix–turn–helix (HTH) structure and bind to the DNA major groove [4, 5]. In contrast, the region C terminal to MYB domain is the highly divergent activation domain, leading to the broad variety of regulatory roles of the MYB gene family [6]. The MYB proteins are classified into different groups according to the number of repeat(s) in the MYB domain. Generally, the MYB members with two repeats are the predominant form found in higher plants, and constitute the R2R3-MYB gene family [7].

Since the first plant MYB gene, *C1,* was isolated, research concerning the identification and functional characterization of the R2R3-MYB gene family has been widely conducted in plants [2, 8]. Numbers R2R3-MYB proteins are involved in the control of many significant physiological and biochemical processes, including the regulation of plant primary and secondary metabolism, the control of plant development, and the participation in response to various biotic and abiotic stresses [2, 9–11]. For example, *BvMYB1* regulates the betalain pathway in beets [12]. *CsMYBF1* from sweet orange controls the hydroxycinnamic acid and flavonol biosynthesis [13]. The MYB protein RCP1 regulates carotenoid pigmentation in *Mimulus lewisii* flowers [14]. *DhMYB1* is involved in the conical cell shape development of the epidermal cells of the *Dendrobium hybrida* flower labellum [15]. The cotton *MYB108* participates in the defense response against *Verticillium dahliae* infection [16]. Moreover, MYB genes are potentially involved in some particular biological traits, such as legume-specific nodulation, wine quality formation in grape and pollinator preference affected by petunia floral UV absorbance [17–19].

Pineapple (*Ananas comosus*) is one of three most important tropical fruits in the world, and is cultivated in almost all the tropical and subtropical areas [20]. Pineapple ranks third in production of tropical fruits, behind bananas and citrus [21], and the world pineapple production has more than sextupled during the past 40 years (4, 127,799 t in 1964 to 25,439,366 t in 2014, http://faostat.fao.org/site/291/default.aspx) according to FAO (Food and Agriculture Organization) statistics, which gives an enormous expectations for this pan tropical crops. Pineapple is a monocotyledonous, perennial, herbaceous plant, and is considered as a good model for studying some particular biological properties, including specialized crassulacean acid metabolism (CAM) photosynthesis, drought tolerance, the collective fruit of multiple fused fruitlets, the occurrence of natural flowering out of season, and the spineless leaves [21–25]. The well-conserved pineapple karyotype and its pivotal phylogenetic position at the base of the order Poales, enable pineapple to be used as a valuable reference for the investigation of monocot evolution [26].

Recent completion of the pineapple genome sequencing allows the genome-wide identification of specific gene families [27]. The dramatic expansion of the R2R3-MYB gene family in higher plants provides a striking example to account for the gene function diversification during evolution. Recently, several genome-wide analyses of R2R3-MYB genes have been conducted in various monocot and dicots species [28–32]. However, little is known about this gene family in pineapple. The available pineapple genomic platform provides an opportunity to reveal the genome-organization of R2R3-MYB gene family in pineapple and to investigate the evolutionary characteristics among different plant species. In this study, we totally identified 94 pineapple R2R3-MYB genes and divided them into 26 subgroups. The comprehensive analysis of the gene structure, gene duplications, chromosome distribution, and phylogeny were further investigated. RNA-Seq data exhibited the expression patterns of *AcMYB* genes in different tissues. Expression profiles under stress and hormonal treatments were evaluated to determine the responses of some *AcMYB* genes to different stresses. This study facilitated the identification of tissue-preferential and stress-related *AcMYB* genes and provided deep insights into the function of R2R3-MYB genes in pineapple.

## Results and discussion

### Identification of 94 *AcMYB* genes and their sequence feature

The amino acid sequence of HMM profile of the Pfam MYB domain (PF00249) was used as a query in BLASTP searches to identify MYB encoding genes presented in pineapple genome. A large number of deduced amino acid sequences (>200 candidates) that contain MYB or MYB-like repeats were obtained. The redundant sequences of candidate genes and MYB genes with incomplete ORFs were excluded for further analysis. The PROSITE and Pfam analyses were subsequently performed to verify the presence of the MYB domains. Finally, a total of 184 non-redundant pineapple MYB proteins were identified, including 94 R2R3-MYB proteins (2R–MYB), 87 1R–MYB proteins and 3 R1R2R3-MYB proteins (3R–MYB). However, no 4R–MYB proteins were identified in our results, which may be due to the incompleteness of the pineapple dataset. The R2R3-MYB proteins represented more than half of the proportion of the total MYB proteins, and thus constituted the largest group of *MYB* genes.

**Fig. 1:**
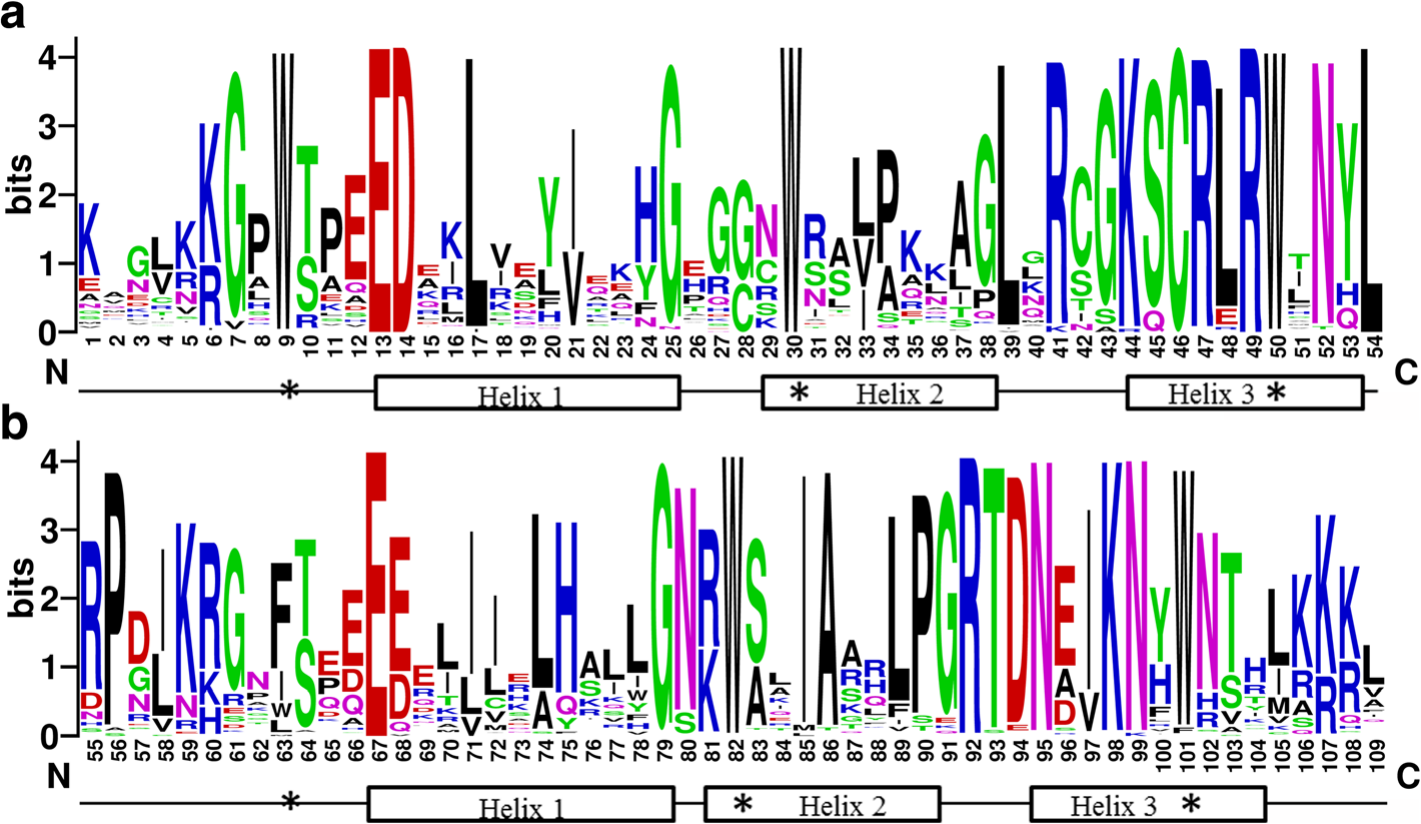
Sequence logos of the R2 (**a**) and R3 (**b**) MYB repeats are based on the full-length alignments of all pineapple R2R3-MYB domains. Multiple alignment analysis of 94 pineapple R2R3-MYB domains was performed with Clustal X. The bit score exhibits the information content for each position in the sequence. The position of the three α-helices are marked (Helix 1 to 3). *Asterisks* indicate the highly conserved tryptophan residues (W) in the MYB domain

Multiple sequence alignment analysis was performed using the amino acid sequences of R2 and R3 repeats (Fig. 1). In general, the isolated pineapple R2R3-MYB proteins contained on average ~ 108 basic residues between the MYB domains. By contrast, the length and amino acid composition were widely divergent in the regions outside of the DNA-binding domain. As compared with those in other plant species, the R2 and R3 MYB repeats of the pineapple R2R3-MYB family contained characteristic amino acids, including a series of evenly distributed and highly conserved Trp residues, which were considered as a landmark of the MYB domain. As with its counterparts in other plant species, the first Trp residue in the R3 repeat was generally replaced by a hydrophobic amino acid, such as Phe (F) or Ile (I). However, substitution by the amino acids Met (M) and Leu (L) were also observed at Trp-62 position of AcMYB proteins.

In addition to the highly conserved Trp residues, Gln-13, Asp-14, Cys-45 in the R2 repeat, Leu-53 in the linker region, and Gln-66 in the R3 repeat were also completely conserved (Additional file 1). The insertion of the Leu residue in the R2 repeat was observed in 73 AcMYB proteins, which was different from that in animal MYB homologs and was an important step for the origin for typical R2R3 MYB proteins [33]. The Leu-38 was substituted with Gln in the R2 repeat of 10 AcMYB proteins, and this replacement may change the DNA-binding specificity of the MYB proteins [34]. The change from Pro-55 to Ser or Ala was observed in four AcMYB proteins, which may have different divergence rate during evolution. This substitution in the linker region may increase the flexibility of the linker and affect the DNA-binding ability [35].

### Genomic location and duplication events among pineapple R2R3-MYB genes

**Fig. 2:**
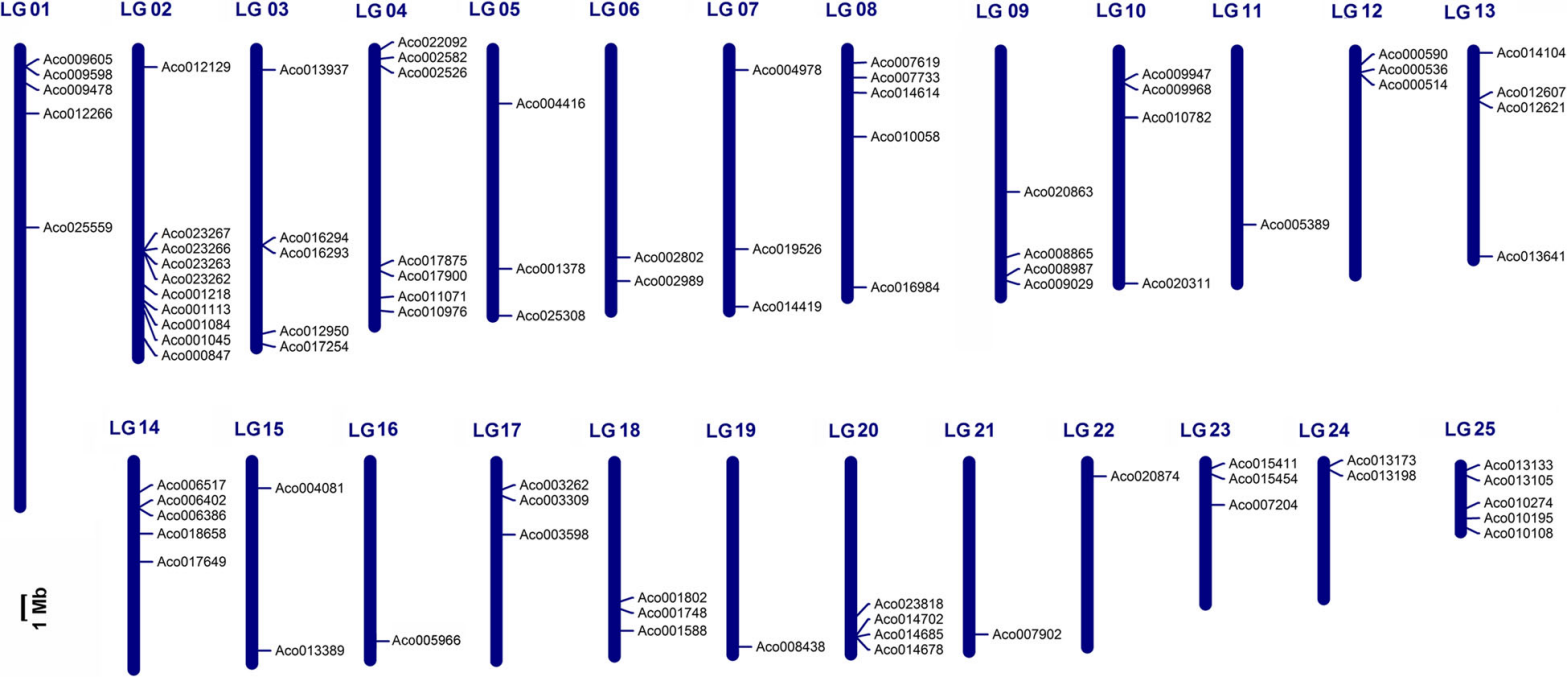
Chromosomal locations of pineapple R2R3-MYB genes. Only 87 R2R3-MYB genes are mapped to the 25 Linkage Groups (LG), while the other seven genes reside on unassembled scaffolds. The chromosomal position of each *AcMYB* gene was mapped according to the pineapple genome. The chromosome number is indicated at the top of each chromosome. The *scale* is in megabases (Mb)

Genome chromosomal location analyses showed that the pineapple R2R3-MYB genes were distributed throughout all 25 Linkage Groups (LG) (Fig. 2). In the currently released sequences, totally 87 MYB genes were mapped to LGs, whereas seven genes were remained on as yet unmapped scaffolds. However, the distribution appeared to be non-random. LG 02 encompassed the largest number of 10 MYB genes followed by 7 on LG 04. In contrast, only one MYB gene was found on LG11, 16, 19 and 21. Substantial clustering of pineapple MYB genes was obvious on several LGs, especially on those with high densities of MYB genes. For example, *Aco023267*, *Aco023266*, *Aco023263* and *Aco023262* were cluster localized on a 14 kb segment on LG 02.

**Fig. 3:**
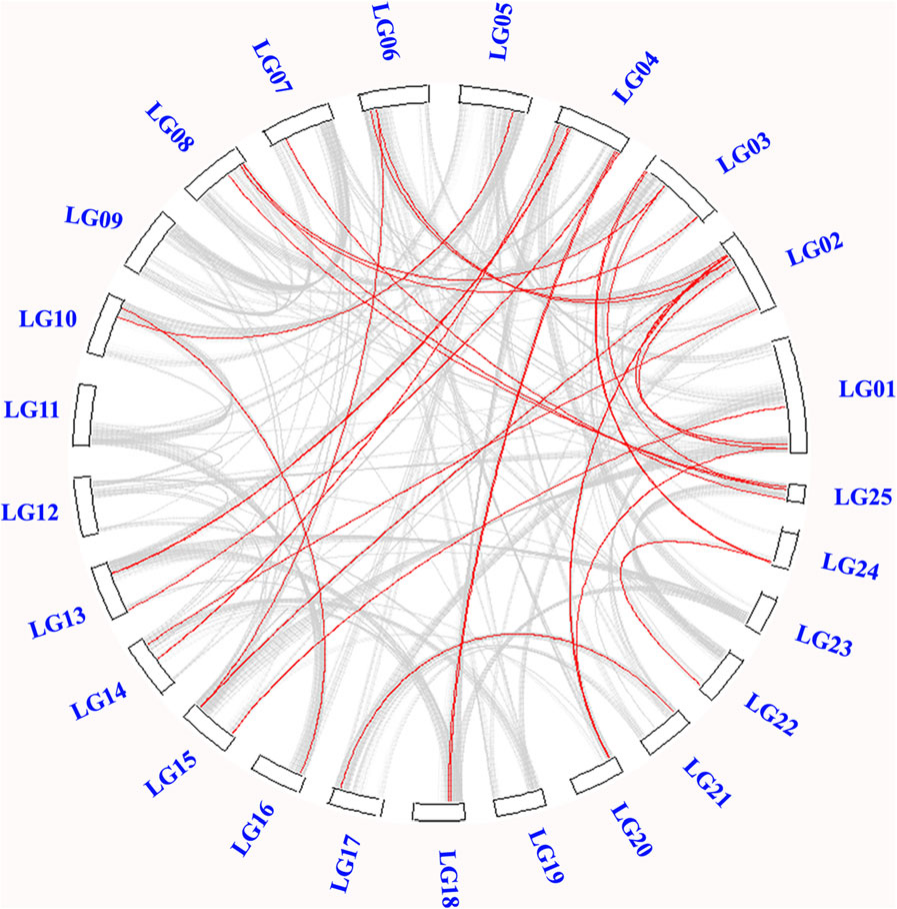
Schematic representations of interchromosomal relationships of the pineapple R2R3-MYB genes. *Gray lines* suggest all synteny blocks in the pineapple genome, and the *red lines* indicate duplicated MYB gene pairs. The chromosome number is indicated at the *top* of each chromosome

Gene duplication has long been recognized to occur throughout plant evolution, and plays an important role in the expansion of the large gene families in plants [36]. To determine the possible relationship between the MYB genes and potential duplication events, the collinearity of the R2R3-MYB gene family in pineapple were identified by BLASTP and MCScanX (Multiple Collinearity Scan) method. Finally, 29 segmental duplication events with 48 MYB genes were identified in pineapple genome (Fig. 3). *AcMYB* genes were located within synteny blocks on almost all LG except LG09, 11, 12, 19 and 22. The intrachromosomal duplication was also observed in the pineapple genome. If two or more MYB genes resided within 20 kb, a gene cluster was defined. In this study, two very closely related pineapple MYB genes (*Aco023266* and *Aco023267*) were physically located near to each other in a syntenic region in LG02, forming one *AcMYB* tandem duplication pair (Additional file 2). All the above segmentally and tandemly duplicated MYB gene pairs had Ka/Ks (non-synonymous/synonymous substitution ratio) values of less than 1, implying that those had evolved under the effect of purifying selection (Additional file 3).

Low tandem and high segmental duplications have been widely observed for the MYB gene family in plants. The gene copies generated by segmental duplication are more often retained in the more slowly-evolving MYB gene family [36], which was supported by a series of recent publications [17, 30, 37]. A large proportion of the segmental duplication events were identified in this study, which was also consistent with the evolutionary pattern of MYB genes. The high segmental duplications indicated that this duplication type likely played a crucial role in the expansion of pineapple MYB gene family.

**Fig. 4:**
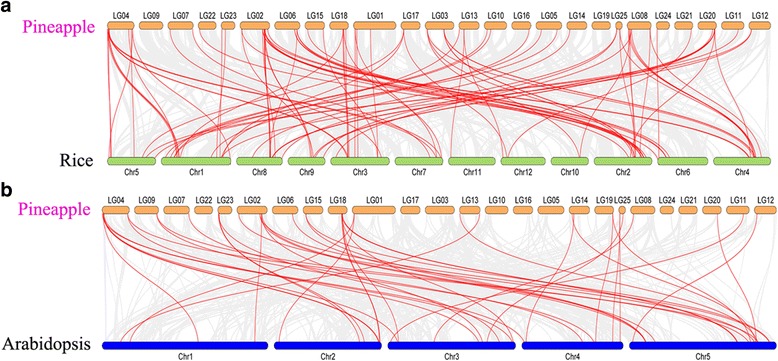
Gene duplication and synteny analysis of R2R3-MYB genes between pineapple and two other model plant species. **a** *Gray lines* in the background indicated the collinear blocks within pineapple and rice genome, while the *red lines* highlight the syntenic MYB gene pairs. **b** *Gray lines* in the background indicated the collinear blocks within pineapple and Arabidopsis genome, while the *red lines* highlight the syntenic MYB gene pairs

To further investigate the potential evolutionary mechanisms of the pineapple R2R3-MYB gene family, we constructed two comparative syntenic maps of pineapple associated with *Arabidopsis thaliana* and *Oryza sativa*, which were belonged to dicotyledon and monocotyledon respectively (Fig. 4). Finally, 44 collinear MYB gene pairs between pineapple and *Arabidopsis* and 92 orthologs between pineapple and rice were identified. The details of the collinear MYB gene pairs were referred in Additional file 4 **.** The number of orthologous events of *AcMYB*-*OsMYB* was far greater than that of *AcMYB*-*AtMYB*, which was consistent with the closer evolutionary distance between pineapple and rice [27]. Significantly, some MYB collinear gene pairs identified between rice and pineapple were anchored to the highly conserved syntenic blocks, which spanning more than 200 genes. However, those between Arabidopsis and pineapple were all located in syntenic blocks that included less than 30 syntenic gene pairs. The pineapple and rice were both in the monocot order Poales, the divergence of pineapple and rice occurred after divergence of Arabidopsis from the common ancestor of monocot and dicot [26]. An extensive level of synteny conservation was found between the pineapple and rice genomes [27], and the higher numbers of orthologous events of *AcMYB-OsMYB* identified in our study may indicate that *AcMYB* genes in pineapple share the similar structure and function with *OsMYB* genes in rice.

### The classification, gene structure and motif composition of pineapple MYB gene family

**Fig. 5:**
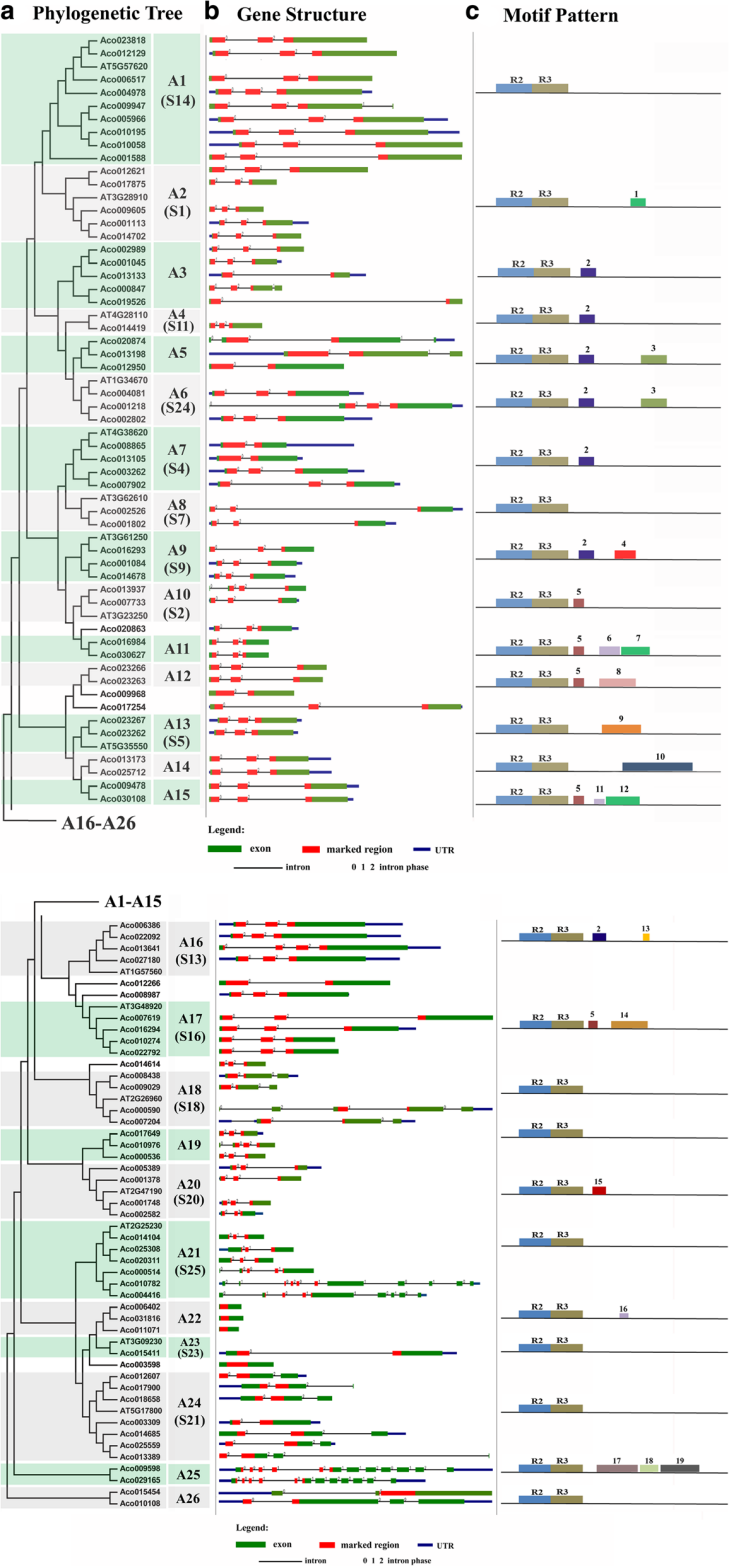
Phylogenetic relationships, gene structure and architecture of conserved protein motifs in R2R3-MYB genes from pineapple. **a** The neighbor-joining (NJ) tree on the left includes 94 R2R3-MYB proteins from pineapple and some representatives from Arabidopsis. The MYB proteins were clustered into 26 subfamilies, sequentially designated as A1 to A26. The subfamily name which was designated as previously reports of AtMYB proteins in Arabidopsis are also marked (Dubos et al., 2010). Six proteins did not fit well into clusters. **b** Exon/intron structures of R2R3-MYB genes from pineapple. Exon(s) and intron(s) are represented by *green boxes* and *black lines*, respectively. The MYB domain(s) are highlighted by *red boxes*, while untranslated region(s) are indicated by *blue boxes*. The number indicates the phases of corresponding introns. **c** Architecture of conserved protein motifs in 26 subfamilies. Each motif is represented by a *number* on the *colored box*

To classify the MYB genes in pineapple, a neighbor-joining (NJ) phylogenetic tree were constructed using the full-length R2R3-MYB protein sequences from pineapple and the model plant Arabidopsis (Additional file 5). Using the Arabidopsis MYB proteins as reference for classification, we subdivided the MYB members of pineapple and Arabidopsis into 26 subgroups (designated A1–A26 in this study) according to the sequence similarity and topology (Fig. 5a). Only representative Arabidopsis MYB genes were shown in the NJ phylogenetic tree. The bootstrap support values for internal nodes were somewhat low, which could be due to the large number of taxa and relatively few informative characters, and this feature was also found in phylogenetic analysis of MYB proteins in other organisms. Most of the large subgroups in our classification were in accordance with those in other plants, and several small subgroups were not retrieved in the previously constructed phylogenetic trees of Arabidopsis MYB proteins. Six pineapple MYB proteins did not fit into any subgroup.

The exon/intron structure analysis for the 94 pineapple MYB genes indicated that most of the coding sequences were disrupted by introns, with exception of four genes (Fig. 5b). The number of introns in the DNA-binding domain (DBD) ranges from zero to five. About 65% of the pineapple MYB genes possessed three exons and two introns in the DBD. Most genes clustered in the same subgroup exhibited similar exon/intron structures. For example, subgroup A22 possessed no introns and the members in A25 contained multiple introns. Additionally, the intron phases within the same subgroup were also conserved in the DBD. The above gene structure analysis provided important evidence for the subgroup designation.

The pineapple MYB protein sequences were subjected to MEME and a total of 19 conserved motifs were identified in the C-terminal regions, which were designated as motif 1 to 19. The details of the 19 motifs were referred in Additional file 6. Most members in the same subgroup shared one or more motifs outside the MYB domain, and high variance was observed between the different subgroup, indicating that the protein architecture was conserved within a specific subgroup (Fig. 5c). The results were similar with those of the phylogenetic analysis, suggesting that the MYB proteins within the same subgroup are likely to share similar functions. Although the functions of most of these conserved motifs have not been elucidated, some of them may play key roles in the transcriptional regulation of target genes.

The similar gene structures and the conserved motifs of MYB gene in the same subgroup, together with the phylogenetic analysis of MYB proteins, could strongly support the reliability of our subfamily classification.

### Comparative phylogenetic analysis of the R2R3-MYB family in six different plant species

**Fig. 6:**
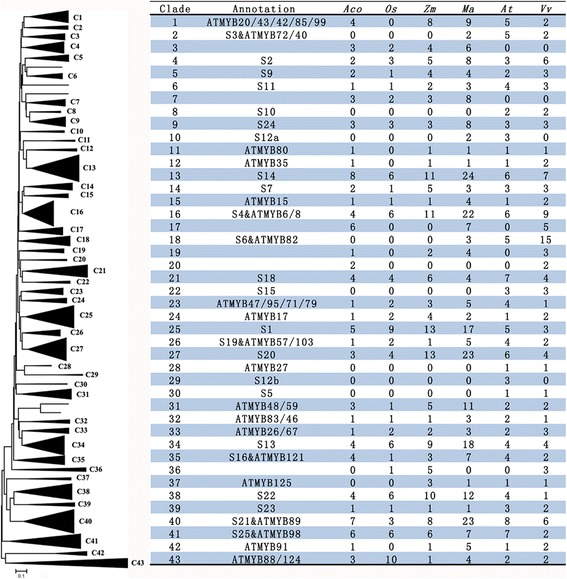
Neighbor-joining (NJ) tree representing relationships among the R2R3-MYB proteins from pineapple (*Aco*), rice (*Os*), maize (*Zm*), banana (*Ma*), Arabidopsis (*At*) and grape (*Vv*). MYB proteins from six species were clustered into 43 subgroups (*triangles*), designated as C1 to C43. The table on the *right* indicated the MYB gene numbers in each subgroup in different species

To gain more insight into the evolutionary characteristics of the MYB gene family, a neighbour-joining phylogenetic tree was constructed using all identified R2R3-MYB protein sequences from pineapple (94), rice (89), maize (157), banana (270), Arabidopsis (126), and grape (122) genomes (Additional file 7). The pineapple was phylogenetically closer to the rice and maize, which were also in the order Poales. Together with the banana, these four species belonged to monocotyledon, and the other two were dicot. The comparative phylogenetic analysis of the R2R3-MYB family in these six different plant species may provide more clues about the evolution history of this gene family. The resulting phylogenetic tree generated 43 subgroups (Fig. 6), which was similar with that obtained when using sequences only from pineapple and Arabidopsis [2].

Phylogenetic analysis revealed that there was not equal representation of the MYB proteins from the six species within given clades. For example, the phylogeny clade C23 included only one MYB proteins from each of four monocotyledon species, while contained three and two MYB proteins from Arabidopsis and grape, respectively. Some clades (i.e., C23, C33, and C4) contained fewer numbers of MYB proteins from pineapple than that from rice and maize, the other two species in the order Polaes. This observation was consisted with our knowledge that the pineapple have not undergone the ρ whole genome duplication event that shared by rice and maize [26]. Most of the clades contained members from all six species, suggesting that the genes within given clades may have already exist in their common ancestral species. However, several clades were found only in some particular species. For instance, two clades (C3 and C7) were present in all four monocots but not in Arabidopsis and grape. By contrast, four clades (C8, C22, C28, and C30) were present only in the two dicots. Remarkably, two clades (C2 and C18) only contained MYB proteins from the three species of Poales but not the other three species. This suggested that the genes in these clades may have specialized roles that were either lost in Polaes or acquired in the other species after divergence from their common ancestor. The pineapple-specific clades and the unequal expansion for pineapple MYB proteins within each clade were not observed, which may be related to the well conserved chromosome karyotype of pineapple [27]. The long term vegetative propagation of pineapple, led to reduced meiosis, may partly explain the observed lower expansion frequency of the pineapple MYB proteins [20].

### Deep transcript abundance profiling of the pineapple R2R3-MYB genes by RNA-seq

**Fig. 7:**
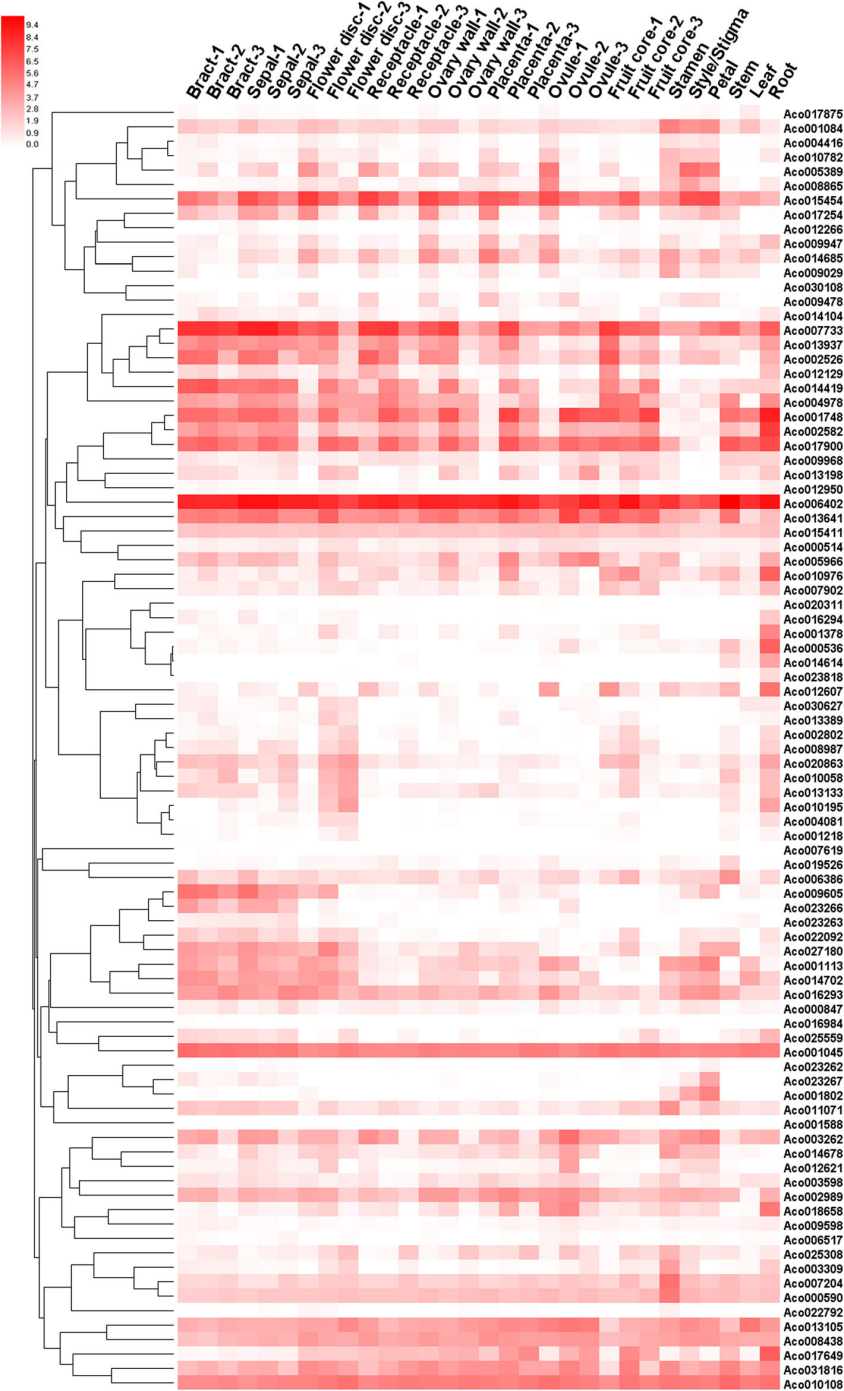
Hierarchical clustering of expression profiles of pineapple R2R3-MYB genes in 30 samples including different tissues and developmental stages. Log_2_(FPKM + 1) values were displayed according to the color code (*top left*)

To understand the temporal and spatial expression patterns of the pineapple R2R3-MYB genes, we analyzed their transcript abundance using transcriptome data of 30 different tissues and developmental stages of pineapple. The transcripts of five MYB genes were not detected in all 30 samples. A lack of expression data may indicate that these were pseudogenes or had special temporal and spatial expression patterns not examined in our libraries. The RNA-seq data was further verified by quantitative real-time PCR experiments which were performed on seven representative samples for 12 selected MYB genes (Additional file 8). A hierarchical cluster analysis was performed using expression data of the other 89 *AcMYB* genes corresponding to the 30 different samples (Fig. 7). In most cases, genes present in the same phylogenetic subgroup exhibited distinct expression patterns, indicating that these genes could perform similar functions in different cell types or in response to different conditions. However, in some cases, closely related MYB genes showed highly similar transcript profiles. This is the case for three members (*Aco009605*, *Aco001113* and *Aco014702*) of subgroup A1, which were also grouped together in the expression cluster.

According to the RNA-seq data, the expression profiles for different *AcMYB* genes varied significantly among the detected samples. 37 MYB genes were expressed in all 30 samples tested (FPKM > 0) and 12 genes showed constitutive expression (FPKM > 2 in all samples). Some genes exhibited preferential expression across the different tissues examined. Thirteen genes in root, one gene in leaf, six genes in stem, nine genes in stamen, one gene in style and three genes in petal showed the highest transcript abundances (Additional file 9). These genes could be involved in the regulation of some biological processes that occurred in the corresponding tissues, and were ideal candidates for further functional analysis. A phylogenetic tree including the complete members of R2R3-MYB proteins from pineapple and Arabidopsis and several function-known rice R2R3-MYB proteins were constructed (Additional file 10). Based on a combination of phylogenetic analysis and RNA-seq data, the crucial functional information of the pineapple MYB genes could be obtained by comparison with those function-known MYB genes from model plants.

*Aco004081* was relatively high expressed in root, and have a close phylogenetic relationship with *AtMYB93*, which was specially expressed in root endodermal cells and regulates lateral root development [38], indicating that this pineapple MYB gene could also be involved in the regulation of root development. Two root-preferentially expressed MYB genes (*Aco017649* and *Aco000536*) shared high sequence similarity with *AtMYB59*, suggesting a similar functional feature in the root growth regulation [39]. Two *AcMYB* genes (*Aco001084* and *Aco014678*) with homology to the two MIXTA-like Arabidopsis MYB genes, showed relatively high expression in stamen, style and petal, indicating that they may have similar functions that regulated the epidermal cell morphogenesis in corresponding floral organs [40]. *Aco001802* was phylogenetically closer to the Arabidopsis flavonol MYB regulators, and showed the preferential expression in petal, suggesting that it could be participated in the regulation of flavonol biosynthesis in petal tissues [41]. Similarly, the petal-preferentially expressed MYB gene, *Aco023267*, might be involved in the regulation of proanthocyanidins biosynthesis in petal [42].

The pineapple fruit belongs to the collective fruit, and the edible part of the fruit consists chiefly of the ovaries, the bases of sepals and bracts and the cortex of the axis [43]. Several MYB genes were probably involved in some biological processes during fruit development and contributed to the formation of pineapple fruit quality characteristics. *Aco014685* had the relatively high expression in various parts of fruitlet including bract, sepal, flower disc, receptacle, ovary wall, placenta and ovule, additionally, the transcript abundances were gradually decreased in developmental stages of these tissues. The gene *Aco014685* was clustered into subgroup A24, and phylogenetically closer to *AtMYB69*, a regulator of the biosynthesis of lignin, xylan and cellulose, participating in secondary cell wall thickening [44]. The pineapple fruit is collectively made up of a number of individual berry-like fruitlets, the bract, sepal and ovary tissues were prominent structures in the mature fruit [20]. The cell walls of these tissues get thinner during the stage from the blossoming inflorescence to mature fruit. Therefore, the gene *Aco014685* could be involved in cell wall regulation during the pineapple fruit development according to its expression pattern and phylogenetic relationship. Similarly, the expression of *Aco012607*, another member of subgroup A24, was gradually reduced during the fruit core development. The fruit core was originated from the inflorescence axis and the firmness was gradually decreased, the gene *Aco012607* may regulate the components of the fruit core cell wall and affect the edible quality of the pineapple fruit.

Additionally, the expression patterns of the pineapple R2R3-MYB genes in transcriptome data from Ming et al. [26] were also provided as a supplement (Additional files 9 and 11), which could provide more comprehensive information for further functional characterization of pineapple MYB genes.

### Expression patterns of *AcMYB* genes under abiotic stresses and hormonal treatments

**Fig. 8:**
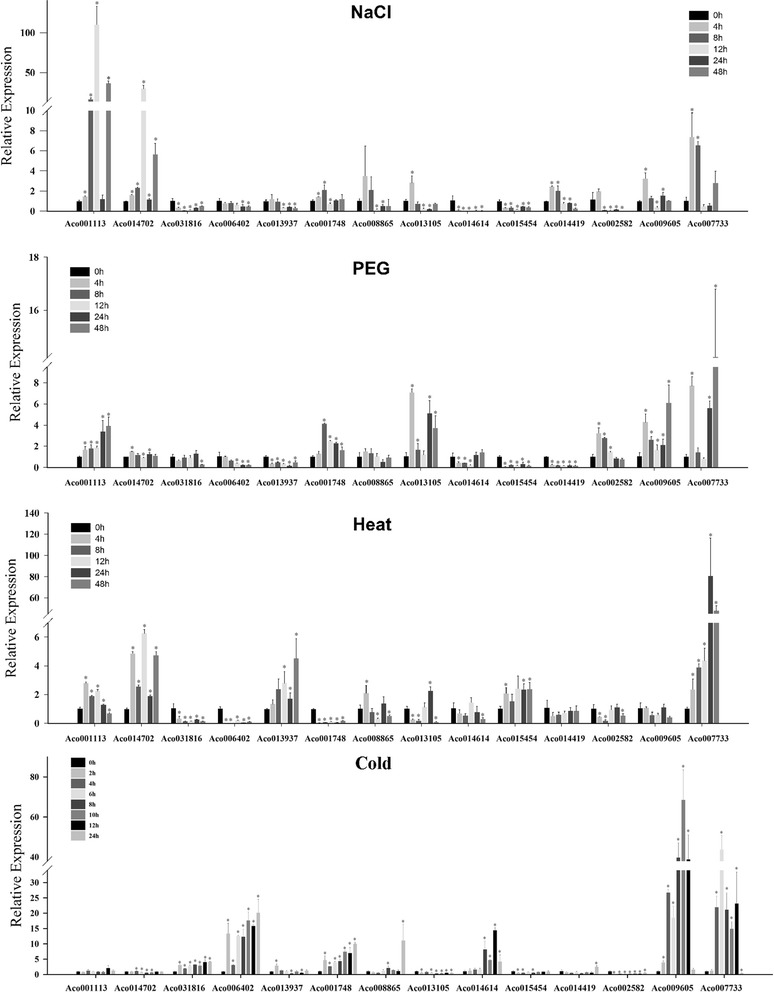
Expression profiles of 14 selected *AcMYB* genes in response to various abiotic stress treatments. Data were normalized to*β-actin* gene and *vertical bars* indicate standard deviation. *Asterisks* (*P* < 0.05, Student’s *t*-test) indicate significant differences compared with the untreated control

**Fig. 9:**
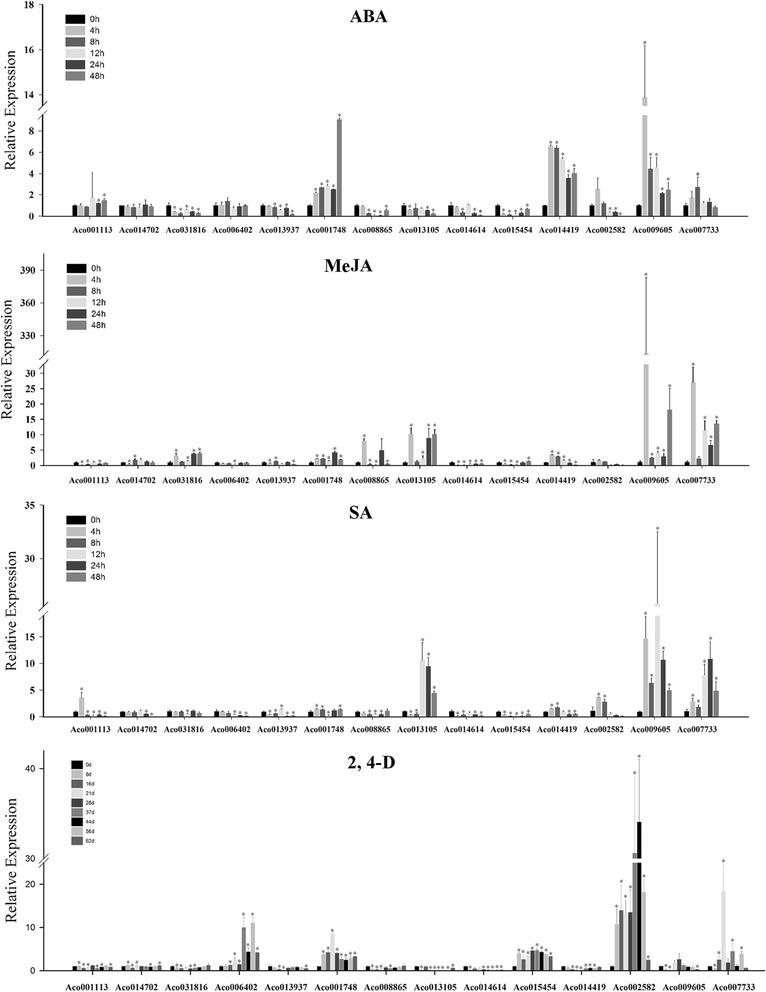
Expression profiles of 14 selected *AcMYB* genes in response to different hormonal treatments. Data were normalized to*β-actin* gene and *vertical bars* indicate standard deviation. *Asterisks* (*P* < 0.05, Student’s *t*-test) indicate significant differences compared with the untreated control

Plants have adopted different strategies to sense, respond and adapt to various biotic and abiotic stresses, which are vital mechanisms for plants to survive in unfavorable environmental conditions [9]. MYB transcription factors have been shown to be essential for the hormonal regulation and stress responses [2]. However, no information is available about pineapple R2R3-MYB genes involved into different stresses. In this study, 14 pineapple R2R3-MYB genes, which were phylogenetically closer to the function-known stress-related MYB proteins in model plants, were selected for further investigation of expression patterns in response to abiotic stress (NaCl, PEG, heat and cold, Fig. 8) and hormonal treatments (ABA, SA, MeJA and 2, 4-D, Fig. 9).The phytohormones adopted in this study were also played important roles in the plant abiotic stress responses [45, 46].

As shown in Figs. 8 and 9, 14 *AcMYB* genes were differentially expressed in response to at least one treatment, most of them could be induced by multiple stress treatments, indicating that they were involved in cross-talk among different signal transduction pathways in response to abiotic stresses. For instance, Aco014614 was only induced by cold treatment, while *Aco001113* was induced by NaCl, PEG and heat treatments. Among of them, *Aco007733* was induced by all eight treatments, suggesting that it was a pleiotropic regulator. However, multiple pineapple MYB genes were simultaneously induced by one treatment. For example, the expression levels of six MYB genes (*Aco031816*, *Aco006402*, *Aco001748*, *Aco014614*, *Aco009605*, and *Aco007733*) were significantly upgraded by cold treatment; four genes (*Aco001748*, *Aco014419*, *Aco009605* and *Aco007733*) were significantly induced by ABA treatment. Additionally, some genes exhibited opposing expression patterns under different stress treatments. For instance, *Aco006402* was significantly up-regulated by cold and 2, 4-D treatments, while down-regulated by heat, PEG and SA treatments.

In accordance with the phylogeny analysis, the selected *AcMYB* genes actually exhibited the stress-responsive expression pattern. However, the stress response types were not completely consistent between *AcMYB* genes and their counterparts. For example, *Aco002582* was induced by ABA, SA, 2, 4-D and PEG treatments, while its orthologs in Arabidopsis, *AtMYB108*, was induced by ABA, JA and NaCl treatments [47]. *OsMPS* was salt-responsive in rice [48], and its phylogenetically closest gene in pineapple, *Ac014614*, was significantly depressed by NaCl treatment. The results implying that the signaling pathways in the plant abiotic stress response were complicated and might be different among the plant species. The genome-wide bioinformatics analysis, combined with the global gene expression profiles analysis under stress treatments, provided a powerful tool for screening valuable candidate genes involved in the abiotic stress responses, and have led to the functional characterization of several stress-related MYB genes, such as *MdoMYB121* in apple and *TaMYB32* in wheat [49, 50]. Take the pineapple MYB gene *Aco006402* as an example, this gene was induced by cold and 2, 4-D treatments and preferentially expressed in root tissues, might be involved in the regulation of stress-induced root-specific biologic processes. Taken together, these above findings might be valuable for improving the environmental resistance of pineapple via the manipulation of the *AcMYB* genes.

## Conclusions

A comprehensive analysis of R2R3-MYB gene family in pineapple was carried out in the present study. A total of 94 full-length R2R3-MYB genes were phylogenetically divided into 26 distinct subfamilies, as supported by the conserved gene structure and the motif composition pattern. They were unevenly distributed among 25 chromosomes in pineapple. Collinearity analysis indicated that the segmental duplication events contributed to the expansion of pineapple MYB gene family. Phylogenomic comparison of R2R3-MYB gene family among pineapple and other five representative species suggested the existence of functional divergence during evolution. The expression analysis led to the identification of tissue-preferential and abiotic stress responsive expression patterns of the *AcMYB* genes. Additionally, putative functions of pineapple MYB genes were assigned based on the phylogenomic results and gene expression data. The results of this work provided information that may facilitate further functional analyses of many newly described R2R3-MYB transcription factors in order to better understanding their biological roles in pineapple.

## Methods

### Identification of pineapple R2R3-MYB family genes

HMM profile of MYB DNA-binding domain (PF00249) was downloaded from Pfam (Protein family: http://pfam.sanger.ac.uk/), and subsequently exploited for the identification of MYB genes from pineapple genome (Join Genome Institute, http://www.phytozome.net) with HMMER 3.0. The default parameters were adopted, and the cutoff value was set to 0.01. To confirm the presence of the core MYB domains, the putative MYB sequences were further examined using Pfam and PROSITE program (http://prosite.expasy.org/scanprosite/). All pineapple R2R3-MYB proteins were manually inspected to ensure that the putative gene models contained two MYB domains.

### Sequence analysis

Multiple sequence alignments of the MYB domains sequences were performed using Clustal X with default parameters. The deduced amino acid sequences in MYB motifs were then adjusted manually using GeneDoc. WEBLOGO (http://weblogo.berkeley.edu/logo.cgi) was used to show up the features of the DNA-binding domains of MYB proteins [51]. The exon/intron organizations of the pineapple MYB genes, including intron distribution patterns, phases and intro-exon boundaries, were analyzed using GSDS (http://gsds.cbi.pku.edu.cn/) tool [52]. To investigate the conserved motifs of pineapple R2R3-MYB proteins, the complete amino acid sequences were subjected to MEME (http://meme.nbcr.net/meme/intro.html) analysis [53]. The optimized parameters of MEME were employed as the following: the maximum number of motifs was set to identify 40 motifs, and the optimum width of each motif was set from 10 to 100 residues.

### Analyses of chromosomal locations and synteny analysis for all *AcMYB* genes

The physical locations of *AcMYB* genes were obtained from the database of pineapple genome. MapChart software was used to draw the location images of *AcMYB* genes [54]. To analyze the duplication pattern for each AcMYB gene, Multiple Collinearity Scan toolkit (MCscanX) was applied and the manipulations followed the operation manual [55]. The Dual Systeny Plotter software (https://github.com/CJ-Chen/TBtools) written by ourselves was adopted to exhibit the synteny relationship of the orthologous MYB genes between pineapple and Arabidopsis as well as that between pineapple and rice. The Ks and Ka were calculated by using DnaSP version 5 software [56].

### Phylogenetic analyses of the AcMYB proteins

The phylogenetic tree of the full-length amino-acid sequences of R2R3-MYB proteins from pineapple and Arabidopsis was constructed using neighbor-joining (NJ) method of MEGA 5.0 [57], with the following parameters: Poisson model; pairwise deletion; and 1000 bootstrap replications. The same method was adopted when constructing the NJ phylogenetic tree encompassing 94 R2R3-MYB proteins from *Ananas comosus* (pineapple), 126 from *Arabidopsis thaliana*, 122 from *Vitis vinifera* (grape), 270 from *Musa nana* (banana), 157 from *Zea mays* (maize) and 89 from *Oryza sativa* (rice). Sequences of the R2R3-MYB proteins from Arabidopsis, maize, rice, and grape were obtained according to the descriptions in the published literatures [2, 19, 30, 37]. R2R3-MYB proteins in banana were identified from the banana genome database using the same method as described above [58].

### Plant materials and treatments

Eight different parts of the pineapple fruit, including bract, sepal, flower disc, receptacle, ovary walls, placenta, ovule, and fruit core, were collected at three fruit development stages from the fruitlet to the mature fruit. Other six tissues including stamen, style, petal, stem, leaf and root were separately collected. Finally, a total of 30 different samples were adopted for the next-step RNA-seq analysis. For investigating the expression pattern of the *AcMYB* genes in response to abiotic stresses, the pineapple (*Ananas comosus* cv. Shenwan) callus tissues and the plantlets were adopted, the samples were kept in good conditions and at the same development stages. For the hormonal treatment experiments, the pineapple callus were subjected to different treatments for 4, 8, 12, 24,48 h in MS liquid medium containing ABA(abscisic acid, 100 μM), SA(salicylic acid, 100 μM), and MeJA (methyl jasmonate, 100 μM), respectively. In the 2, 4-D (2, 4-dichlorophenoxyacetic acid) treatment experiment, the callus samples were cultured on the MS solid medium containing 4 mg/L 2, 4-D, and collected at 8,16, 21, 28, 37, 44, 56 and 62 days. The sample were subjected to salinity and drought stress by transferring the callus to 150 mM NaCl and 15% PEG (PEG6000) solution, respectively, for 4, 8, 12, 24 and 48 h. For the heat and cold stress treatments, the tissue culture plantlets were kept at 40 and 4 °C, respectively. The samples were collected at 2, 4, 6, 8, 10, 12, 24 h in cold stress treatment and 4, 8, 12, 24, 48 h in heat stress treatment. All samples were immediately frozen in liquid nitrogen and store at −80 °C until used.

### RNA isolation and quantitative real-time RT-PCR

Total RNA extractions of the collected samples were carried out using TRIZOL reagent (Takara), with the procedures described previously [59]. RNA quality was monitored by gel electrophoresis and the measurement of A260/A280 ratio. For cDNA synthesis, 1 μg RNA was reverse-transcribed using the HiScript® II 1st Strand cDNA Synthesis Kit (Vazyme) according to the manufacturer’s procedure. Primers were designed for real-time quantitative PCR (qRT-PCR) using Primer Express 3.0 software (Applied Biosystems), and the primer sequences were shown in detail in Additional file 12. QRT-PCR was conducted on Roche Lightcyler® 480 instrument using SYBR Green Master Mix (Vazyme). The reactions were carried out with the following cycling profile: 95 °C for 30 s, followed by 40 cycles of 95 °C/10 s, 60 °C/30 s. Each reaction was performed in biological triplicates, and the relative gene expression values were calculated using the 2^-△△CT^ method. The pineapple *β-actin* gene was used as the internal reference gene.

### Expression analyses of *AcMYB* genes by RNA-seq

Total RNA of the aforementioned 30 collected samples, including various tissues and organs at different developmental stages as well as specialized tissues such as stamen and style, were chosen for further RNA-seq library construction (Details in Additional file 13). The transcript abundance of pineapple R2R3-MYB genes was calculated as fragments per kilobase of exon model per million mapped reads (FPKM). The log_2_(FPKM + 1) from the RNA-seq data were subjected to hierarchical clustering using Cluster 3.0, and the results were visualized by Java TreeView [60].

## Additional files

Additional file 1: Multiple alignment of the amino acid sequences of 94 pineapple R2R3-MYB domains. (TIFF 4472 kb)

Additional file 2: Segmentally and tandemly duplicated *AcMYB* gene pairs. (XLSX 12 kb)

Additional file 3: Ka/Ks calculation of the duplicated pineapple R2R3-MYB gene pairs. (XLSX 11 kb)

Additional file 4: One-to-one orthologous relationships between pineapple and Arabidopsis as well as that between pineapple and rice. (XLSX 16 kb)

Additional file 5: Phylogenetic tree of the R2R3-MYB proteins from pineapple and Arabidopsis based on neighbor-joining method using MEGA 5.0 software. The numbers beside the branches represent bootstrap support values (>50%) from 1000 replications. (PDF 12 kb)

Additional file 6: Consensus sequences of the group specific motifs. (XLSX 11 kb)

Additional file 7: Neighbor-joining tree representing relationships among 94 R2R3-MYB proteins from pineapple, 89 from rice, 157 from maize, 270 from banana, 126 from Arabidopsis and 122 from grape. MYB members from each species are marked by different shapes (▽, pineapple; ◇, rice; ▲, maize; ●, banana; ◆, Arabidopsis; ○, grape). The MYB proteins were clustered into 43 subgroups and group designations are marked on the right. The numbers beside the branches represent bootstrap support values (>50%) from 1000 replications. (PDF 1142 kb)

Additional file 8: Expression analysis of 12 MYB genes in seven representative samples by qRT-PCR. Data were normalized to *β-actin* gene and vertical bars indicate standard deviation. The corresponding FPKM values were listed. The Pearson correlation coefficient (*r*) between the qRT-PCR and RNA-seq (FPKM) data and the associated *p* value were shown accordingly. (TIFF 1084 kb)

Additional file 9: The RNA-seq data of *AcMYB* genes in different tissues and developmental stages. (XLSX 89 kb)

Additional file 10: Phylogenetic tree of the 94 R2R3-MYB proteins from pineapple, 126 from Arabidopsis and 12 well-characterized rice MYB proteins (with red solid circle) based on neighbor-joining method using MEGA 5.0 software. The numbers beside the branches represent bootstrap support values (>50%) from 1000 replications. (PDF 341 kb)

Additional file 11: Expression profiles of pineapple R2R3-MYB genes in different samples. Expression profiles of pineapple R2R3-MYB genes in the RNA-seq data derived from the pineapple green tip (A) and white base (B) leaf tissues at 2-h intervals over a 24-h period [26]. (C) Expression profiles of pineapple R2R3-MYB genes in the RNA-seq data derived from different tissues and fruit development stages. Log_2_(FPKM + 1) values were displayed according to the color code (top left). (TIFF 1742 kb)

Additional file 12: The primers used in this study. (PDF 162 kb)

Additional file 13: Details about the transcriptome data used in this study. (PDF 169 kb)

## Abbreviations

AcMYB: Pineapple (*Ananas comosus*) R2R3-MYB
AtMYB: *Arabidopsis (Arabidopsis thaliana)* R2R3-MYB
OsMYB: Rice (*Oryza sativa*) R2R3-MYB
