# Supplementary figures and images for: Genome-wide organization and expression profiling of the R2R3-MYB transcription factor family in pineapple (Ananas comosus)
Source: BMC Genomics. 2017 Jul 1;18:503. doi: 10.1186/s12864-017-3896-y (PMC5494133; doi:10.1186/s12864-017-3896-y)

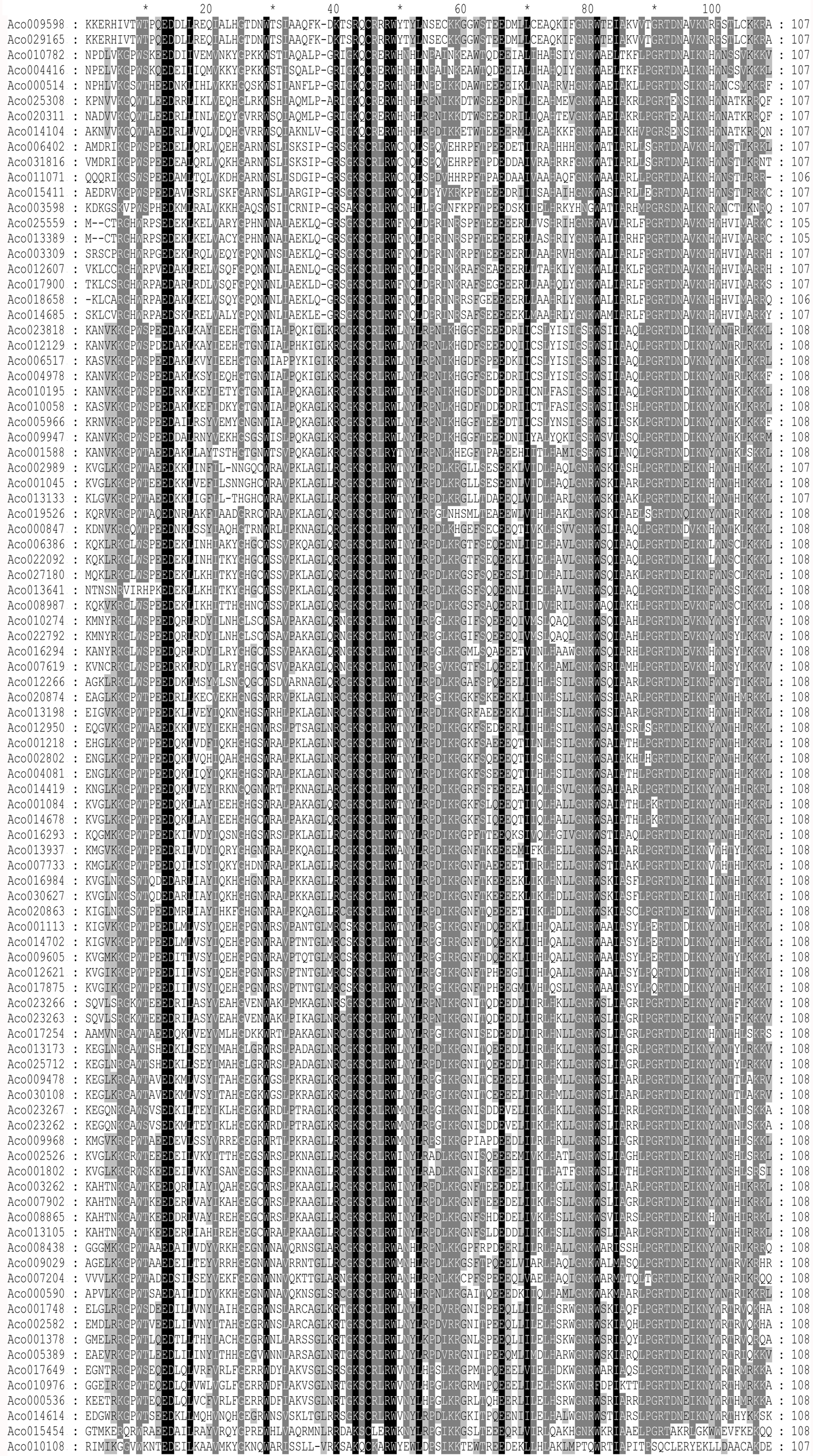

Supplement: Supplementary file 1 — Multiple alignment of the amino acid sequences of 94 pineapple R2R3-MYB domains. (TIFF 4472 kb) [file 12864_2017_3896_MOESM1_ESM.tif]

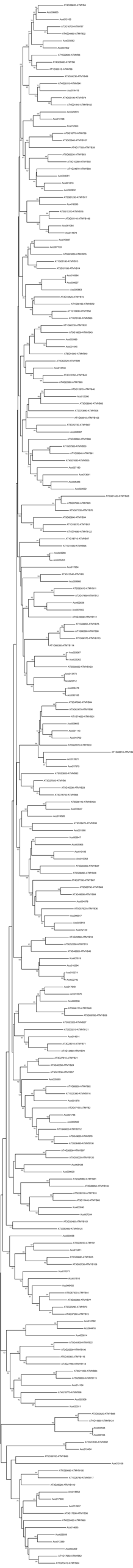

0.1

Supplement: Supplementary file 5 — Phylogenetic tree of the R2R3-MYB proteins from pineapple and Arabidopsis based on neighbor-joining method using MEGA 5.0 software. The numbers beside the branches represent bootstrap support values (>50%) from 1000 replications. (PDF 12 kb) [file 12864_2017_3896_MOESM5_ESM.pdf]

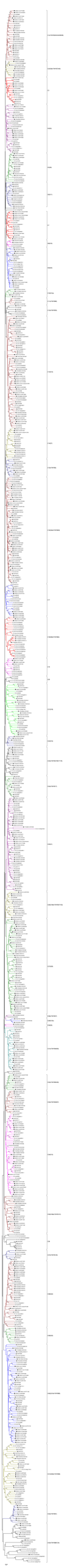

Supplement: Supplementary file 7 — Neighbor-joining tree representing relationships among 94 R2R3-MYB proteins from pineapple, 89 from rice, 157 from maize, 270 from banana, 126 from Arabidopsis and 122 from grape. MYB members from each species are marked by different shapes (▽, pineapple; ◇, rice; ▲, maize; ●, banana; ◆, Arabidopsis; ○, grape). The MYB proteins were clustered into 43 subgroups and group designations are marked on the right. The numbers beside the branches represent bootstrap support values (>50%) from 1000 replications. (PDF 1142 kb) [file 12864_2017_3896_MOESM7_ESM.pdf]

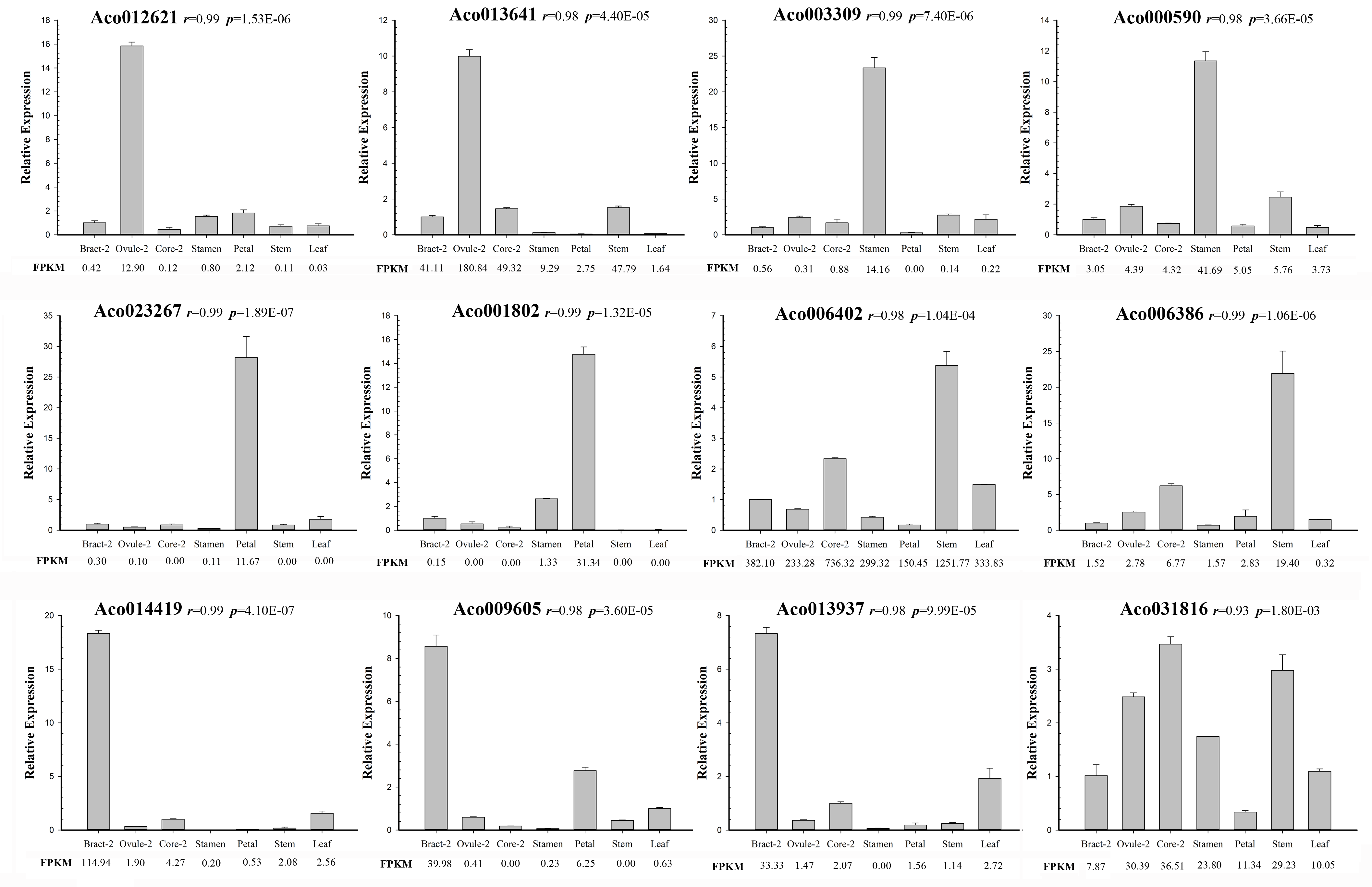

Supplement: Supplementary file 8 — Expression analysis of 12 MYB genes in seven representative samples by qRT-PCR. Data were normalized to β-actin gene and vertical bars indicate standard deviation. The corresponding FPKM values were listed. The Pearson correlation coefficient (r) between the qRT-PCR and RNA-seq (FPKM) data and the associated p value were shown accordingly. (TIFF 1084 kb) [file 12864_2017_3896_MOESM8_ESM.tif]

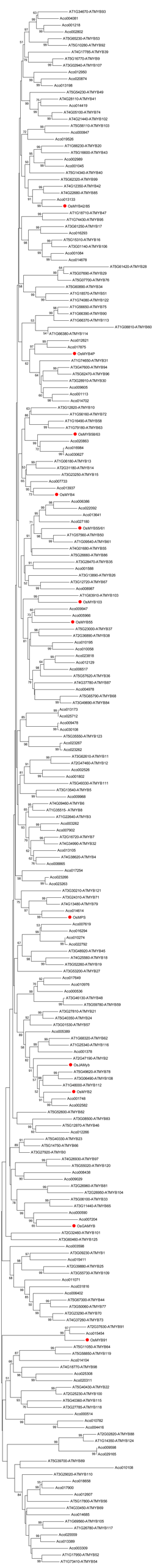

Supplement: Supplementary file 10 — Phylogenetic tree of the 94 R2R3-MYB proteins from pineapple, 126 from Arabidopsis and 12 well-characterized rice MYB proteins (with red solid circle) based on neighbor-joining method using MEGA 5.0 software. The numbers beside the branches represent bootstrap support values (>50%) from 1000 replications. (PDF 341 kb) [file 12864_2017_3896_MOESM10_ESM.pdf]

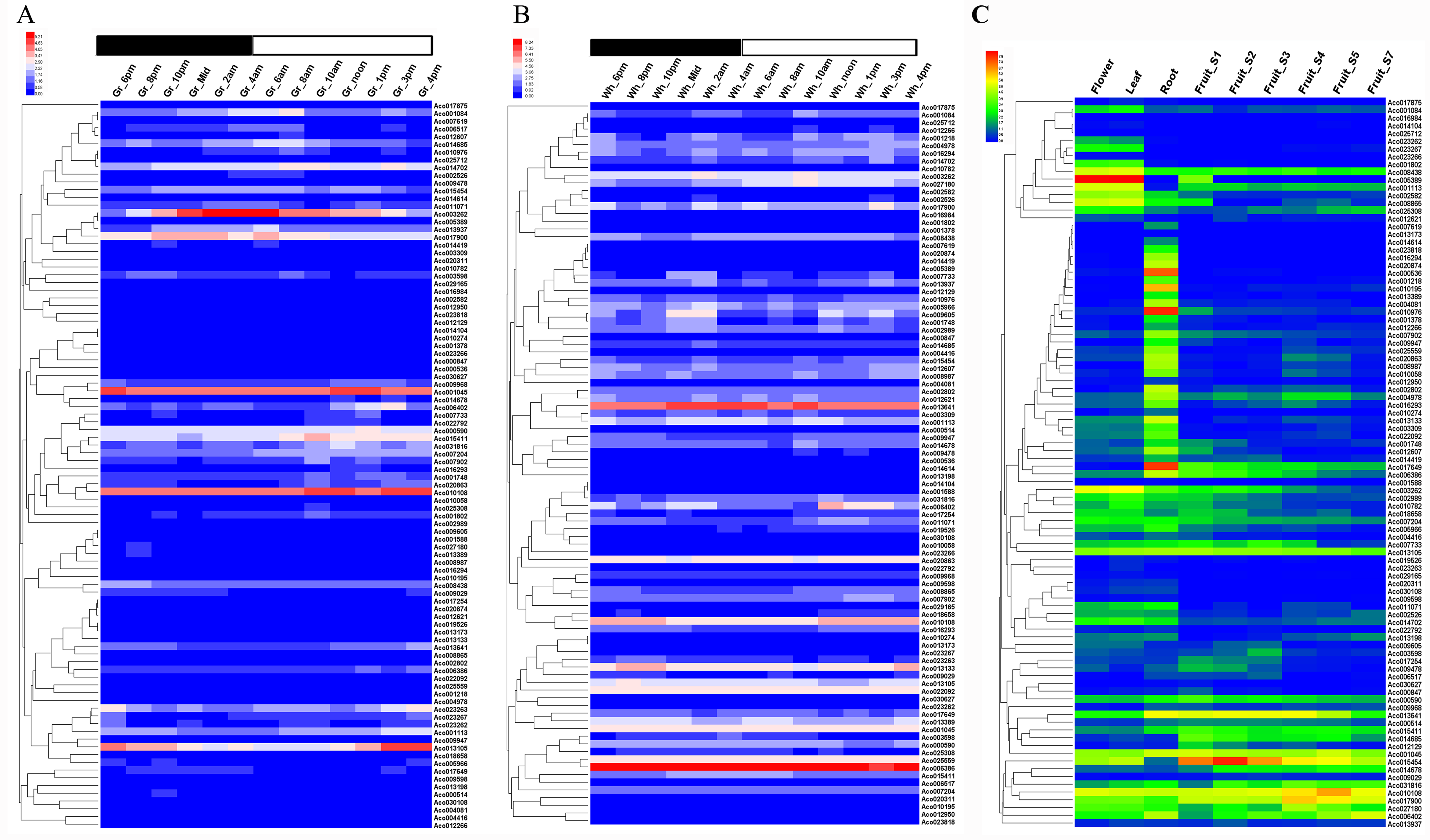

Supplement: Supplementary file 11 — Expression profiles of pineapple R2R3-MYB genes in different samples. Expression profiles of pineapple R2R3-MYB genes in the RNA-seq data derived from the pineapple green tip (A) and white base (B) leaf tissues at 2-h intervals over a 24-h period [26]. (C) Expression profiles of pineapple R2R3-MYB genes in the RNA-seq data derived from different tissues and fruit development stages. Log2(FPKM + 1) values were displayed according to the color code (top left). (TIFF 1742 kb) [file 12864_2017_3896_MOESM11_ESM.tif]
