# Supplementary material for: Genome-wide organization and expression profiling of the R2R3-MYB transcription factor family in pineapple (Ananas comosus)
Source: BMC Genomics. 2017 Jul 1;18:503. doi: 10.1186/s12864-017-3896-y (PMC5494133; doi:10.1186/s12864-017-3896-y)
Supplement: Supplementary file 12 — The primers used in this study. (PDF 162 kb) [file 12864_2017_3896_MOESM12_ESM.pdf]

Additional file 12 The primers used in this study.

Primer name	Primer sequences	Primer name	Primer sequences
OAco009605-F	TCACAAGCCAACCCTACAGC	OAco001748-F	CTATGAAACGCAACGAGACGA
QAco009605-R	CGGCGAGTTCTTCTTCCATC	QAco001748-R	CCTGCCAATTTCCTAAGTCCA
QAco001113-F	CCACAACCTACGCATCAAACA	QAco002582-F	CGGTGTCTTCTGCTTGTTTCA
QAco001113-R	TTGGGCTTGGTTGAGTTCCT	QAco002582-R	CGGTAGTGATTCCGTCCAGTT
QAco014702-F	GTCAGGAACCCACCAAAGAAG	QAco014419-F	CCAAGCATACTTCAAATGGTCA
QAco014702-R	CCGAACCGCCTTACTACCA	QAco014419-R	GGTGTCGATAAGACGGAGTCAA
QAco031816-F	TGGTGATGGATCGGATAAAGG	QAco008865-F	AACCACTGGAACACGCACAT
QAco031816-R	CCTCCCAGGTATGGATTTGC	QAco008865-R	TAACAAAAGAAGCGGCGAAA
QAco006402-F	CGTCGCCTTTCCAGTTCAG	QAco013105-F	TCCAAGAAGAAGAGGAGGCG
QAco006402-R	ATGTAGCTCCGCACCTCGTC	QAco013105-R	ACAACACTTGAAACAGAAGAGCC
QAco013937-F	GCTTCTCGTCCTCAGTTGCC	QAco014614-F	CTTTGTGGGGAAACAGATGGT
QAco013937-R	GGTGGTCGCAAAACAATCAT	QAco014614-R	CGTCCGCCAGTAGTTCTTGAT
QAco007733-F	TGCGAACCACGAAAAGAGG	QAco015454-F	GAGGGAGAAGATGGAGGAGATC
QAco007733-R	GATGATAAGCCGCTGAACGA	QAco015454-R	CTTCTGCTCCTTGGCATCG
QAco000590-F	CAAGAAACCCTATCTCGCCC	QAco006386-F	CAGAGCCATAGAAGCCCAGTT
QAco000590-R	AAGTGCCGTTATTACCTCGTTG	QAco006386-R	GCTGCTGTGGCTGCTGATT
QAco013641-F	TCTACGACAGCGGCATCTTT	QAco012621-F	CCTCCCTCAAAGGACAGACAA
QAco013641-R	GGTATTCGGACCATTTGAGG	QAco012621-R	CAGGTGGGTGTTCCAGTAGTTC
QAco003309-F	TTCACGGATAGCTCACAACCA	QAco023267-F	GATGATACCGCAAAGCCAAAC
QAco003309-R	CGAAGGCAATACACCGAAAG	QAco023267-R	GGAGGAAGCAAGGAAGAAGATG
QAco001802-F	CAGAGCCATAGAAGCCCAGTT	Qβ-actin-F	CTGGCCTACGTGGCACTTGACTT
QAco001802-R	GCTGTGGCTGCTGATTCTTC	Qβ-actin-R	CACTTCTGGGCAGCGGAACCTTT
