# Supplementary material for: Genome-wide organization and expression profiling of the R2R3-MYB transcription factor family in pineapple (Ananas comosus)
Source: BMC Genomics. 2017 Jul 1;18:503. doi: 10.1186/s12864-017-3896-y (PMC5494133; doi:10.1186/s12864-017-3896-y)
Supplement: Supplementary file 13 — Details about the transcriptome data used in this study. (PDF 169 kb) [file 12864_2017_3896_MOESM13_ESM.pdf]

Additional file 13: Details about the transcriptome data used in this study

Total RNA extraction of the collected samples was carried out using TRIZOL reagent (Takara). The RNA concentrations were measured using Qubit 2.0 Fluorometer and NanoDrop 2000 spectrophotometer, whereas the quality was assessed using the Agilent 2100 Bioanalyzer (Agilent Technologies, Santa Clara, CA, USA). The RNA-seq library was constructed using the Illumina TruSeq Stranded RNA Sample Preparation kit (RS-122-2001) and then sequenced using the Illumina HiSeq4000 platform in paired-end 125-nt mode, which were performed at the Beijing's Biomarker Technologies Co. Ltd. Twenty-seven samples including stamen, style, petal, and eight tissues (bract, sepal, flower disc, receptacle, ovary walls, placenta, ovule, and fruit core) with three different development stages, were sequenced with one time. The leaf, stem and root samples were sequenced with three biological replications.

The detailed transcriptome data processing workflow was indicated by **Figure A1**. The raw reads from each sample were listed in **Table A1**, and all raw reads were filtered by using Trimmomatics [1] to remove sequencing adapters and low quality bases (the clean reads were also listed in **Table A1**). To ensure low contamination of rRNA reads in all data, a home-made pipeline were built, firstly bwa was applied to map all reads to SILVA rRNAs [2] database and then removed all mapped reads with a perl script. After this, the resultant high quality reads were aligned against the pineapple F153 genome [3] with STAR aligner [4]. Htseq-count was then applied to summary raw counts of all genes. A home-made toolkit (https://github.com/CJ-Chen/TBtools) was used to transform raw counts to FPKM matrix. Raw data from each sample was submitted to the Sequence Read Archive (SRA) at NCBI under Project ID **PRJNA382449**. Additionally, the transcriptome data was also available on http://pineappledb.xyz/PineappleExp/.

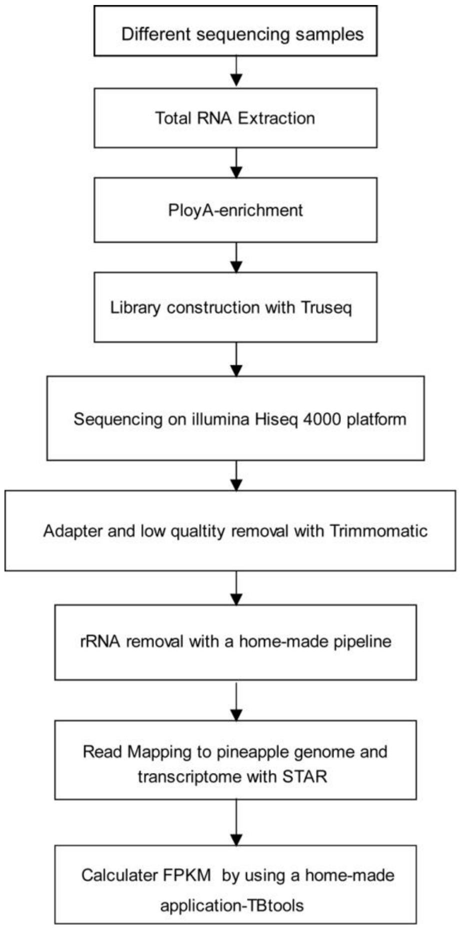

Figure A1 Summary of the transcriptome data processing workflow

Table A1 The raw reads and clean reads from each sequencing sample

Sample name	Raw Reads	Clean reads
Bract-1	18,443,768	17,771,876
Bract-2	15,474,382	14,912,854
Bract-3	18,139,467	17,476,312
Sepal-1	13,285,569	12,758,597
Sepal-2	14,972,512	14,419,860
Sepal-3	12,410,456	11,912,081
Flower disc-1	13,922,625	13,418,429
Flower disc-2	14,554,931	14,035,736
Flower disc-3	16,778,938	16,228,487
Receptacle-1	4,286,364	4,080,360
Receptacle-2	28,944,774	27,848,656
Receptacle-3	20,351,132	19,638,896
Ovary wall-1	19,497,864	18,389,240
Ovary wall-2	6,217,814	5,749,654

Ovary wall-3	15,543,953	15,085,028
Placenta-1	18,829,769	18,216,118
Placenta-2	13,395,923	12,971,552
Placenta-3	23,542,483	22,770,490
Ovule-1	16,798,554	16,093,363
Ovule-2	17,110,029	16,541,806
Ovule-3	15,952,787	15,364,306
Stamen	15,802,470	14,594,351
Style/Stigma	15,287,802	14,758,920
Core-1	13,877,978	13,365,135
Core-2	14,600,200	12,801,514
Core-3	17,458,466	11,816,827
Petal	21,290,450	20,581,810
Stem-1	17,499,133	16,912,254
Stem-2	16,760,738	15,570,431
Stem-3	15,218,631	12,941,291
Leaf-1	16,144,444	15,122,522
Leaf-2	15,856,100	15,197,883
Leaf-3	18,422,227	17,661,113
Root-1	18,234,623	17,446,765
Root-2	18,982,701	18,111,539
Root-3	18,922,999	18,114,790

Reference

1. Bolger AM, Lohse M, Usadel B. Trimmomatic: a flexible trimmer for Illumina sequence data. Bioinformatics 2014:btu170.
2. Pruesse E, Quast C, Knittel K, Fuchs BM, Ludwig W, Peplies J, Glöckner FO. SILVA: a comprehensive online resource for quality checked and aligned ribosomal RNA sequence data compatible with ARB. Nucleic Acids Res 2007, 35(21):7188-7196.
3. Ming R, VanBuren R, Wai CM, Tang H, Schatz MC, Bowers JE, Lyons E, Wang M-L, Chen J, Biggers E. The pineapple genome and the evolution of CAM photosynthesis. Nat Genet 2015, 47(12):1435-1442.
4. Dobin A, Davis CA, Schlesinger F, Drenkow J, Zaleski C, Jha S, Batut P, Chaisson M, Gingeras TR. STAR: ultrafast universal RNA-seq aligner. Bioinformatics 2013, 29(1):15-21.
